# Gummatous neurosyphilis in an elderly patient in the Australian outback: a case report

**DOI:** 10.1186/s13256-021-03153-1

**Published:** 2021-11-09

**Authors:** Nilesh Anand Devanand, Krishnaswamy Sundararajan

**Affiliations:** Intensive Care Unit, Level 4, Royal Adelaide Hospital, Port Road, Adelaide, SA 5000 Australia

**Keywords:** Syphilis, Neurosyphilis, Gumma, CSF VDRL

## Abstract

**Introduction:**

Neurosyphilis is an infection caused by the spirochete *Treponema pallidum*, which causes infiltration and thickening of brain meninges. Despite being an Old World disease, the rates of infection continue to rise. This clinical challenge involves early and accurate diagnosis, as neurosyphilis masquerades with various clinical symptoms and is often missed during initial presentation to the hospital. A comprehensive history and clinical examination are essential to detect suspicious cases early for further cerebrospinal fluid examination and neuroimaging. Patients treated with benzylpenicillin for a specific duration often show promising clinical and cognitive improvement, thus emphasizing the need for constant vigilance in our day-to-day practice.

**Case presentation:**

A 77-year-old Caucasian gentleman presented to our hospital repeatedly with multiple episodes of presyncope and cognitive impairment. He also demonstrated bilateral deafness, tabes dorsalis, and left sixth cranial nerve palsy. His cerebrospinal fluid examination showed a nonreactive venereal disease research laboratory test, and magnetic resonance imaging of the brain revealed a gumma.

**Conclusion:**

The diagnosis of neurosyphilis in the elderly requires a combination of clinical vigilance and a high index of suspicion, along with multimodal investigations, including cerebrospinal fluid examination and brain imaging.

## Introduction

Syphilis is a sexually transmitted disease that has been present for centuries. It is caused by the spirochete *Treponema pallidum* and is transmitted horizontally through sexual contact or vertically from mother to baby. Its disease is broadly categorized into four stages: the *primary stage*, characterized by the development of a painless ulcer known as a chancre on the genitalia; the *secondary stage*, often seen with disseminated disease, which predominates with cutaneous manifestations; and a late early and late *latent phase*, whereby obvious clinical symptoms have resolved, and laboratory investigations are key to making a diagnosis. The *tertiary stage* is characterized by the presence of *gumma*, a rubbery noncontractile tumor that mainly presents on the skin but can occur in other organs, including the brain. In Australia, the national update on sexually transmitted diseases demonstrated an increase of 146% in syphilis notifications from 2014 to 2018, predominantly among women [1].

Neurosyphilis, often a late sequelae of untreated syphilis, presents with multiple neurocognitive signs and symptoms, including syncope, memory loss, dementia, vision and hearing impairment, cranial nerve involvement, peripheral neuropathy, and, rarely, cerebral gummas. The variety of clinical presentations combined with the lack of a gold standard test has made the diagnosis often challenging. It has raised many controversies surrounding the utility of cerebrospinal fluid (CSF) assays for disease confirmation. So far, there are limited Australian data on neurosyphilis, especially with cerebral gummas.

Neurosyphilis is often diagnosed with a combination of clinical and CSF findings. However, the interplay between neuroinvasion and inflammatory markers is complex. While a CSF venereal disease research laboratory (VDRL) test is thought to be highly specific, its sensitivity is debatable, with most published papers in the current era citing a sensitivity in the range of 50–60% [2].  Additionally, CSF pleocytosis and elevated protein counts are often ambiguous in their diagnostic value and do not add to the overall disease detection.

## Case presentation

Our patient is a 77-year-old frail gentleman with type 2 diabetes mellitus on oral hypoglycemic medications who lives with his daughter, requiring support with daily living activities. His past medical history is significant for hypertension, chronic kidney disease, and gout. He is a former smoker and denies recent alcoholism. Prior to his monogamous relationship with his partner, he vaguely recalls having unprotected sexual relations in his teens, almost 50 years ago. He denies being treated for any sexual transmitted diseases (STDs), and confirms nil significant family history for STDs.

He mobilizes with a four-wheel walker, and presented to the hospital with vomiting, presyncope, and an unwitnessed fall without loss of consciousness.

He reports almost daily postural dizziness and has had several short admissions in the past for recurrent presyncope and falls. He denies other systemic complaints.

His history is significant for bilateral hearing impairment, previously attributed to a vestibular disease and a possible past syphilis infection which remains unknown within the local STD registry. It is uncertain whether treatment was ever commenced. A baseline VDRL test was unavailable.

On examination, his vitals were stable with demonstrable postural hypotension. A neurological examination demonstrated a right eye relative afferent pupillary defect, left eye abducens nerve palsy, and a high stepping gait of the right foot. A review of his symptoms was otherwise unremarkable, except for a prolonged PR interval (200–220 milliseconds) on his electrocardiogram (ECG).

His routine blood biochemistry was as expected, and syphilis serology detected *Treponema pallidum* particle agglutination (TPPA) as well as previously undetected rapid plasma reagin (RPR) titers, giving rise to uncertainty as to whether the syphilis infection was prior or current. A lumbar puncture displayed clear CSF within normal pressures with a high protein count of 2.59 mg/dl and an elevated CSF white cell count at 12 × 10^6^/L (Table 1). In addition, the CSF syphilis nucleic acid antigen (NAA) test was negative, CSF fluorescent treponemal antibody (FTA) was minimally reactive, and CSF TPPA and VDRL were both nonreactive.

**Table 1:** Results of cerebrospinal fluid (CSF) test prior to commencement of antibiotics

CSF test	Results	Reference range
Biochemistry		
Protein	2.59 g/L	0.15–0.45 g/L
Glucose	4.6 mmol/L	2.7–4.2 mmol/L
CSF examination		
Description	Clear and colorless	
Microscopy		
Leukocytes (×10^6^/L)	12	< 5 × 10^6^/L
Polymorphs (%)	4	
Mononuclear cells (%)	96	
Erythrocytes (×10^6^/L)	4	< 5 × 10^6^/L
India Ink	No *Cryptococcus* seen	
Gram stain	No organism seen	
Culture	No growth after 48 hours	
Syphilis serology		
CSF FTA	Minimally reactive	
CSF TPPA	Nonreactive	
CSF VDRL	Nonreactive	

CSF: Cerebrospinal fluid, FTA: Fluorescent Treponemal Antibody absorption test, TPPA: Treponemal pallidum particle agglutination assay, VDRL: Venereal Disease Research Laboratory test

His echocardiogram revealed normal ventricular function without valvular pathologies. Alarmingly, an inpatient Holter test detected a 6-second sinus pause that was emergently addressed with the insertion of a pacemaker within 24 hours and a brief postprocedural cardiac care unit admission for observation.

**Fig. 1:**
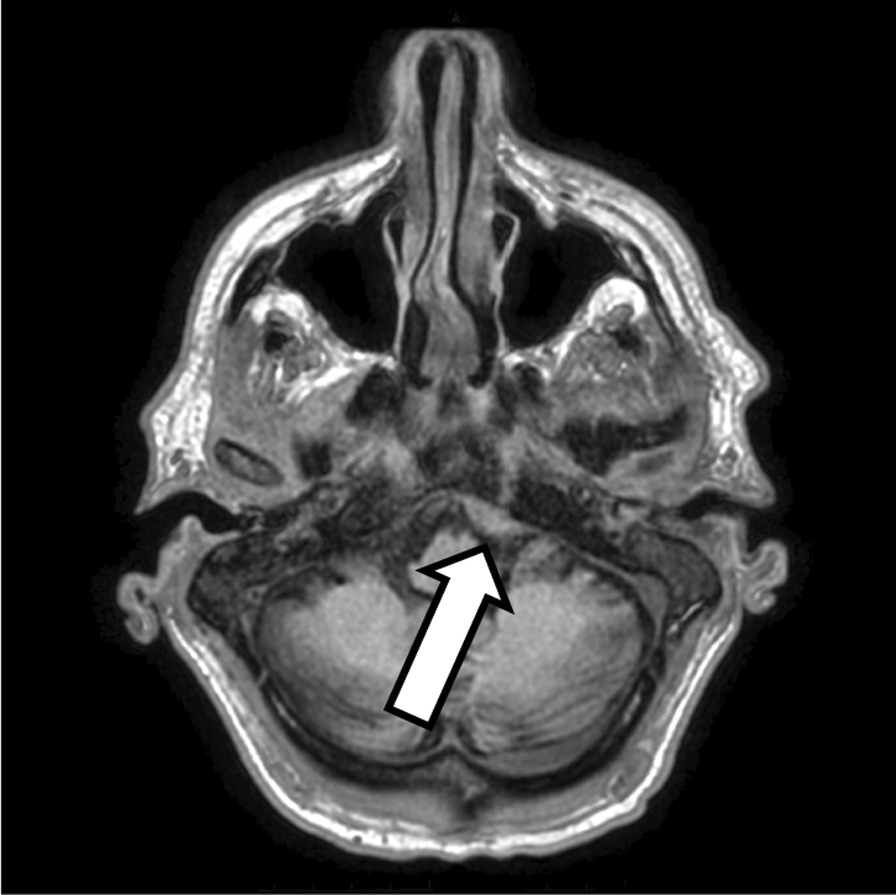
T1-weighted brain MRI demonstrating a thickening of the meninges that was initially considered a meningioma but subsequently re-diagnosed as a gumma (left, arrow)

A non-contrast computerized tomography (CT) scan of the brain showed thickening of the meninges at the left clivus, resembling a meningioma, and this was further followed up by a magnetic resonance imaging (MRI) of the brain. The MRI reported similar findings, with the addition of possible chronic mastoiditis. As a consequence of this significant finding, a multidisciplinary collaboration between geriatrics, neurosurgery, and radiology was undertaken. It was concluded beyond reasonable doubt that the MRI was consistent with a radiological diagnosis of gumma (Fig. 1). With clinical consensus, the final diagnosis of gummatous neurosyphilis was made.

A cognitive assessment was attempted while the investigation (pre-treatment) was ongoing but was limited by hearing and vision impairment. A baseline Mini-Mental State Examination (MMSE) was attempted but was prematurely stopped because of the patient’s poor comprehension of the examination questions. An assessment of delirium was also limited for the same reason.

**Fig. 2:**
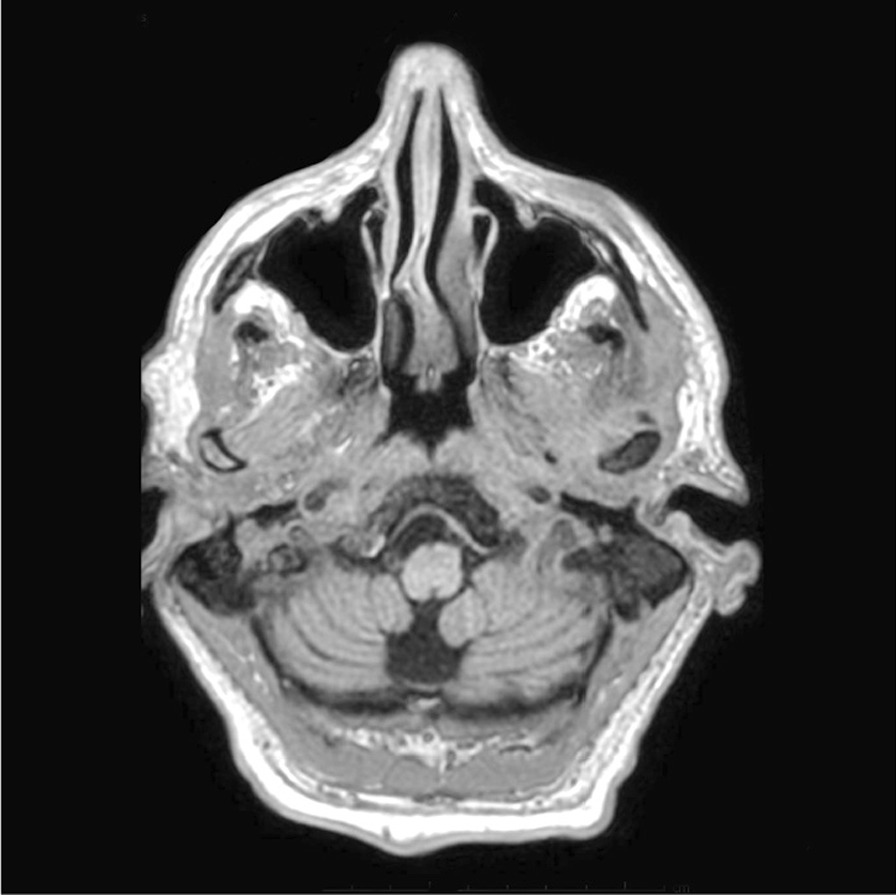
Following therapy with intravenous benzylpenicillin, a follow-up brain MRI 3 months later displayed a pronounced reduction in the size of the gumma (right)

He was commenced on 1.8 million units of intravenous benzylpenicillin, 4-hourly for 15 days, and was sent to the Geriatric Medical Unit for ongoing care and physiotherapy. On completing his antibiotic regime, he made a satisfactory recovery—notably, with an improvement in his cognition from severe to mild cognitive impairment (MMSE 22/30) post-treatment and an appreciative reduction in the size of his gumma on the repeat MRI scan (Fig. 2).

Despite negative CSF *Treponema pallidum* serology, which is known to have low sensitivity and may thus contribute to false negative cases, the poignant history of bilateral deafness, the presence of a sixth cranial nerve pathology, an Argyll Robertson pupil, a tabes dorsalis gait, and an extremely high CSF protein with a gumma on the brain MRI collectively diagnosed this patient as having gummatous neurosyphilis.

## Discussion

Neurosyphilis, an infectious complication of the central nervous system caused by the spirochete *Treponema pallidum* continues to increase in prevalence [3] but remains challenging to diagnose quickly and accurately [4] because of multiple non-specific symptoms that can manifest at different stages of the disease.

In the early stages, infiltration into the CSF, meninges, and blood vessels is often seen. The late presentation usually involves brain parenchyma and the spinal cord, necessitating the need for CSF examination [5].

Neurosyphilis often masquerades with non-specific symptoms, and unclear laboratory investigations frequently lead to delayed and inaccurate diagnosis. Especially in elderly patients because of confounding influences and age-related cognitive decline.

The current criteria for diagnosing neurosyphilis depends on a combination of reactive serological test results including CSF antibody and TPPA test, white cell and protein count, as well as a reactive CSF-VDRL with or without clinical symptoms. Suspicion of neurosyphilis arises when the CSF white blood cell count is higher than 20 × 10^6^ cells/L or a CSF VDRL test result is reactive [6].

Neurosyphilis should also be considered in patients presenting with neuropsychiatry symptoms. Including neuroimaging as a mode of investigation often helps to elucidate dural thickening, as the disease can affect the meninges [7].

## Conclusion

While neurosyphilis remains a disease of the Old World, it continues to be prevalent. This case demonstrates that, with a high index of clinical suspicion, coupled with timely investigations, accurate interpretation, and targeted therapy in a multidisciplinary approach, we could significantly reduce the burden of this indolent disease and improve patient care in resource-constrained environments.

## Data Availability

Data are available on request.

## Abbreviations

CSF: Cerebrospinal fluid
FTA: Fluorescent treponemal antibody
MMSE: Mini-Mental State Examination
NAA: Nucleic acid antigen
RAPD: Relative afferent pupillary defect
RPR: Rapid plasma reagin
TPPA: *Treponema pallidum* particle agglutination
VDRL: Venereal disease research laboratory
